# Giant Muscle Invasive Dermatofibroma Clinically Mimicking a Malignant Tumor

**DOI:** 10.1155/2019/4503272

**Published:** 2019-03-28

**Authors:** Hideyuki Kinoshita, Takeshi Ishii, Hiroto Kamoda, Toshinori Tsukanishi, Sumihisa Orita, Kazuhide Inage, Seiji Ohtori, Tsukasa Yonemoto

**Affiliations:** ^1^Department of Orthopedic Surgery, Chiba Cancer Center, 666-2 Nitonacho, Chuo-ku, Chiba 260-8717, Japan; ^2^Department of Orthopaedic Surgery, Graduate School of Medicine, Chiba University, 1-8-1 Inohana, Chuo-ku, Chiba 260-8670, Japan

## Abstract

Dermatofibromas are common benign fibrohistiocytic lesions, usually appearing as slow growing firm dermal nodules with a predilection for the extremities (mostly the lower legs). They are found mostly in middle-aged women and are usually smaller than 2 cm in diameter. Giant dermatofibromas exceeding 5 cm in diameter are rare. In recent years, reports have suggested a relationship between the primary size of dermatofibromas and rates of local recurrence and metastases after surgery. This relationship is however debated. The present report describes the case of a giant muscle invasive tumor in a 51-year-old female patient who presented with a large ulcerated mass in the right upper arm. The tumor appeared clinically malignant, measuring approximately 12 cm × 6 cm in size, with ulceration and invasion of surrounding muscle. Wide resection of the tumor was performed with myocutaneous flap-plasty. Histopathological examination showed evidence of a dermatofibroma. No recurrence, metastases, or other complications were noted at 5 years after surgery. The present case demonstrates that although dermatofibromas are essentially benign, they may present with atypical features including large size, ulceration, and muscle invasion, clinically mimicking malignant tumors.

## 1. Introduction

Dermatofibromas, also known as benign fibrous histiocytomas, are common harmless cutaneous nodules arising from reactive fibroblastic proliferation of unknown etiology. They are most frequently found in the extremities and are more common in women [1]. They usually present as red-brown or yellow-brown papules on the limbs, particularly in the thighs and lower legs, measuring less than 2 cm in diameter [2]. The tumor is typically located in the skin, involving the subcutaneous fat, dermis, and epidermis, without invasion to surrounding muscles. However, atypical clinical findings have been reported in recent years, including giant tumor size, associated ulceration, and the occurrence of metastases [3, 4]. These atypical tumors may be clinically difficult to distinguish from malignant tumors, leading to a possibility of misdiagnoses. Appropriate histological evaluation is an important tool to prevent misdiagnoses, allowing distinction between benign dermatofibromas and malignant tumors such as dermatofibrosarcoma protuberans (DFSP) [5].

Here we present a case of giant muscle invasive dermatofibroma, clinically suspicious of a malignant tumor. Successful wide resection was performed, and the patient remains healthy without recurrence for 5 years.

## 2. Case Presentation

**Figure 1:**
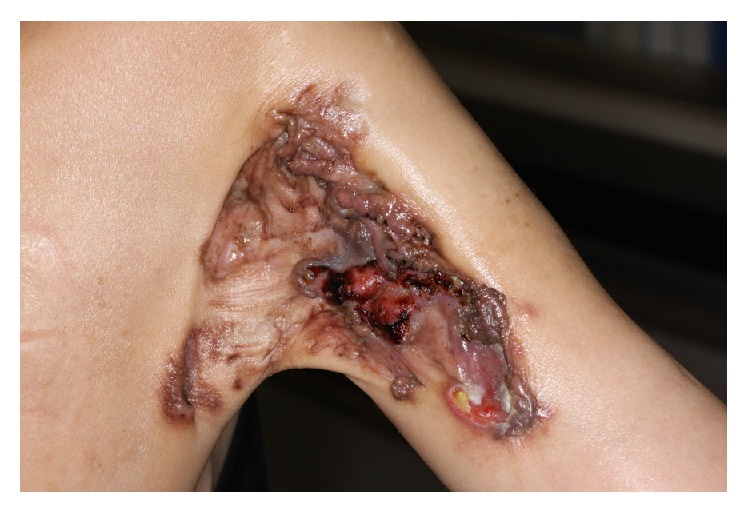
The large nodulo-ulcerative plaque with bleeding seen on frontal view; located at the back of the right upper arm, measuring 12 cm × 6 cm.

**Figure 2:**
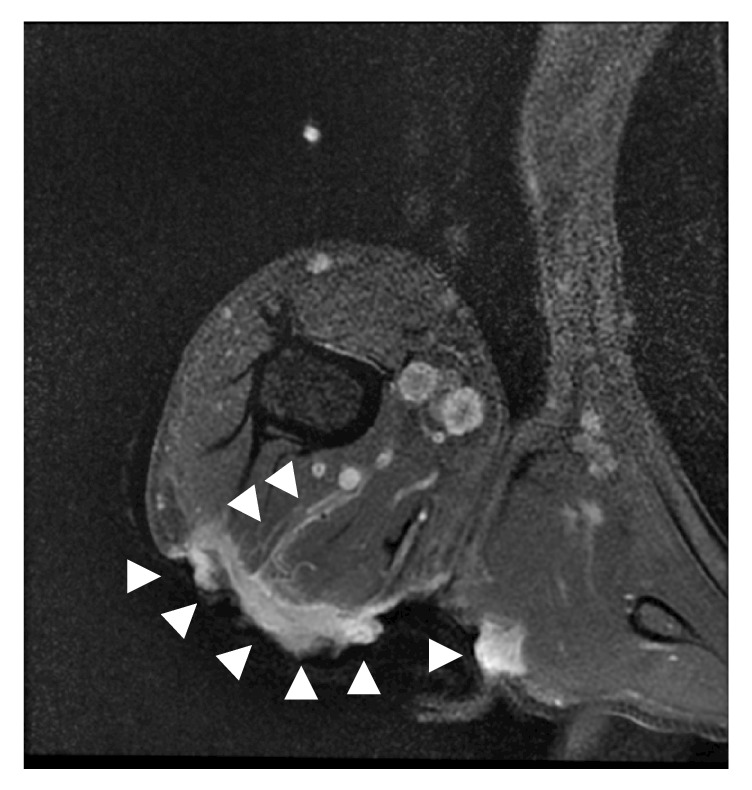
Axial view of the dermatofibroma (arrowhead) on magnetic resonance imaging; invasion to the deltoid and triceps brachii muscles is seen.

**Figure 3:**
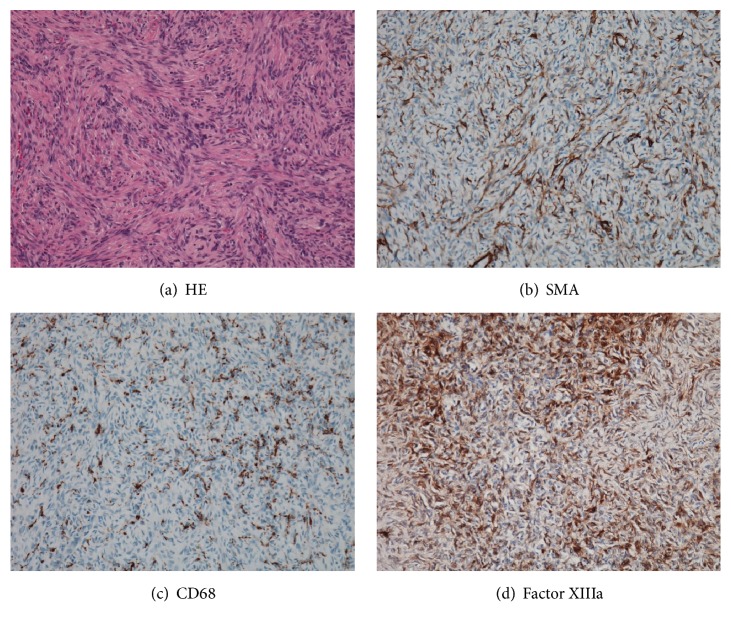
(a) Proliferation of spindle cells with vague storiform growth pattern and basal cell hyperplasia of overlying epidermal cells noted on hematoxylin and eosin staining. (b–d) Immunohistochemical stain showing positivity for (b) SMA, (c) CD 68, and (d) factor XIIIa.

A 51-year-old female patient presented to our orthopedic outpatient department with a rapidly enlarging ulcerated right upper arm swelling which was growing gradually over the past six months. She recalled that it had been a pea-sized lesion and had remained the same size over the past 30 years. No history of preceding episodes of trauma or local irritation was found. The initially asymptomatic lesion had recently become painful and pruritic with slight bleeding. On examination, a large nodulo-ulcerative plaque was noted on the right upper arm, measuring approximately 12 cm × 6 cm in size (Figure 1). Magnetic resonance imaging (MRI) revealed a mass measuring 12 cm × 6 cm × 1.5 cm, invading the deltoid and triceps brachii muscles (Figure 2). Examination of the needle biopsy specimen demonstrated the proliferation of spindle cells in a storiform arrangement in the dermis. Results of tests for CD34 and factor VIII were negative, suggesting the possibility of a benign tumor. However, the clinical findings, which included muscle invasion, large tumor size, and rapid enlargement, suggested the possibility of malignancy, prompting the need for wide resection and myocutaneous flap-plasty. Intraoperatively, the tumor was found to have invaded the deltoid and triceps brachii muscles, as well as the cutaneous and subcutaneous tissue. Wide resection was achieved with a 3 cm margin. A latissimus dorsi musculocutaneous flap was needed to close the skin defect. The postoperative period was uneventful. Histopathological examination following hematoxylin and eosin stain revealed spindle cell proliferation with a vague storiform growth pattern and basal cell hyperplasia in the overlying epidermal cells (Figure 3(a)). The tumor tested negative for CD 34 on immunohistochemical staining but tested positive for SMA, CD 68, and factor XIIIa (Figures 3(b)–3(d)). Mitoses were inconspicuous in the lesion, and the Ki 67 labeling index was also low, at 1 to 2%. These findings were consistent with a diagnosis of dermatofibroma. In 5 years of follow-up, the patient did not experience any recurrence, metastases, or other complications.

## 3. Discussion

Dermatofibromas are common, usually benign, soft-tissue tumors often found on the extremities, particularly the lower limbs. They typically present as asymptomatic, firm, slow-growing, red-brown, or yellow-brown dome-shaped nodules, measuring between a few millimeters to 1 to 2 cm in diameter [2]. In the current case, the tumor measured 12 cm × 6 cm, which was large compared to a typical dermatofibroma. On attaining large sizes, these lesions are clinically called giant dermatofibromas. In their series of eight cases reported in 1994, Requena et al. reported the features that have been widely accepted to define “giant” dermatofibromas. These include (a) size of 5 cm or larger, (b) pedunculated lesions, (c) benign biological behavior despite their size, and (d) the same histopathological characteristics as conventional dermatofibromas [6]. Although the large dermatofibroma in the present case enlarged rapidly clinically mimicking malignancy, it did not meet all the mentioned defining criteria. Therefore, according to the mentioned criteria, the lesion in the present case could not be defined as a typical “giant dermatofibroma.” Dermatofibromas are known to be benign lesions. It was believed that although they could recur locally, they lacked the capacity to metastasize [7]. However, in recent years, in addition to local recurrence, some dermatofibromas have been reported to have metastasized during postoperative follow-up. In their case series, Leona et al. reported series that metastases occurred several months to years after diagnosis, and the lungs, lymph nodes, soft tissues, and liver were the principal sites of metastasis. They also reported that the primary tumor size was related to the incidence of local recurrence and metastases after surgery [4]. Dermatofibromas are usually located in the skin, involving the subcutaneous fat, dermis, and epidermis. Although muscle invasive dermatofibromas have been reported in post-excision cases, muscle invasion prior to surgery is rare [8], and this finding is usually indicative of malignancy. In the present case, the tumor was very large, had overlying ulceration, invaded muscle, and was hemorrhagic, clinically suggestive of malignancy. Despite a needle biopsy diagnosis suggestive of a benign tumor, wide resection was performed with a musculocutaneous flap, based on a clinical suspicion of malignancy. Five years after surgery, the patient is well and has a healthy-looking scar with no signs of recurrence and metastasis, despite the giant preoperative tumor size and muscle invasion.

The differential diagnoses of dermatofibromas include other benign lesions such as nodular fasciitis, neurofibromas, and leiomyomas, as well as malignant tumors such as DFSP [5]. On immunostaining, dermatofibromas typically test negative for the CD 34 antibody (positive in 85% of DFSP) and positive for SMA, CD 68, and factor XIIIa [9]. In the case described, the immunohistochemical reactions were suggestive of a dermatofibroma, and mitoses were not conspicuous; Ki 67 labeling index was also low, suggestive of a benign tumor such as dermatofibroma. Therefore, a combination of these immunohistochemical reactions is necessary for an accurate diagnosis.

In conclusion, the present report describes the case of a giant muscle invasive dermatofibroma, which was clinically suspected to be malignant owing to its appearance. Giant dermatofibromas that invade the muscle may recur or metastasize after surgery. In these cases, adequate surgery, thorough histopathological examination, and careful follow-up are essential for better outcomes.
